# Supplementary material for: A stochastic epigenetic switch controls the dynamics of T-cell lineage commitment
Source: eLife. 2018 Nov 13;7:e37851. doi: 10.7554/eLife.37851 (PMC6245732; doi:10.7554/eLife.37851)
Supplement: Supplementary file 1. — Each antibody specifies the cell populations targeted and their corresponding reference figures. [file elife-37851-supp1.docx]

Supplementary File 1: List of antibodies used for magnetic bead protocols, flow cytometry analysis, and sorting. Each antibody specifies the cell populations targeted and their corresponding reference figures.

| **Antibody** | **Usage** |
| --- | --- |
| Anti-mouse CD8α Biotin (clone 53-6.7) | Thymic DP and SP depletion. Figure 6A (Thymic DNs analysis), Figure 6B (FL DNs analysis) |
| Anti-mouse TCRβ Biotin (clone H57-597) | Thymic DP and SP depletion |
| Anti-mouse TCRγδ Biotin (clone GL3) | Thymic DP and SP depletion |
| Anti-mouse Ter119 Biotin (clone TER-119) | Thymic DP and SP depletion, Bone marrow depletion, Fetal liver depletion. Figure 2 (BM-derived DNs sort) |
| Anti-mouse NK1.1 Biotin (clone PK136) | Thymic DP and SP depletion, Bone marrow depletion, Fetal liver depletion. Figure 2 (BM-derived DNs sort) |
| Anti-mouse Gr-1 Biotin (clone RB6-8C5) | Thymic DP and SP depletion, Bone marrow depletion, Fetal liver depletion, Splenocyte depletion. Figure 2 (BM-derived DNs sort) |
| Anti-mouse CD11c Biotin (clone N418) | Thymic DP and SP depletion, Bone marrow depletion, Fetal liver depletion, Splenocyte depletion. Figure 2 (BM-derived DNs sort) |
| Anti-mouse CD11b Biotin (clone M1/70) | Thymic DP and SP depletion, Bone marrow depletion, Splenocyte depletion. Figure 2 (BM-derived DNs sort) |
| Anti-mouse CD19 Biotin (clone 1D3/6D5) | Bone marrow depletion, Fetal liver depletion, Splenocyte depletion. Figure 2 (BM-derived DNs sort) |
| Anti-mouse CD3ε Biotin (clone 145-2C11) | Bone marrow depletion. Figure 2 (BM-derived DNs sort), Figure 3-figure supplement 1 (Total Thymocytes analysis) |
| Anti-human/mouse B220 Biotin (clone RA3-6B2) | Bone marrow depletion |
| Anti-mouse F4/80 Biotin (clone BM8) | Fetal liver depletion |
| Anti-mouse CD4 Biotin (clone GK1.5) | Figure 6A (Thymocyte DNs analysis), Figure 6B (FL DNs analysis) |
| Anti-human/mouse CD44 eFluor 450 (clone IM7) | Figure 1C (ETP-DN3, CD4/CD8 analysis), Figure 3B (ETP-DN3 analysis), Figure 3D (Thymocyte DNs sort and analysis), Figure 6A (Thymocyte DNs sort and analysis), Figure 6B (FL DNs sort and analysis), Figure 3-figure supplement 1 (Depleted Thymocytes analysis), Figure 3-figure supplement 2 (Depleted Splenocytes analysis), Figure 3-figure supplement 3 (Total Splenocytes analysis) |
| Anti-mouse CD25 Brilliant Violet 510 (clone PC61) | Figure 1C (ETP-DN3 analysis), Figure 3B (ETP-DN3 analysis), Figure 3D (analysis), Figure 6B (FL DNs sort), ­­ Figure 3-figure supplement 1 (Depleted Thymocytes analysis) |
| Anti-mouse CD117 (cKit) APC-eFluor 780 (clone 2B8) | Figure 1C (ETP-DN3 analysis), Figure 3B (ETP-DN3 analysis), Figure 3-figure supplement 1 (Depleted Thymocytes analysis) |
| Anti-mouse HSA eFluor 450 (clone M1/69) | Figure 1C (DP analysis), Figure 3B (DP analysis), Figure 6A (Total Thymocytes sort and analysis), Figure 3-figure supplement 1 (Total Thymocytes analysis) |
| Anti-mouse CD4 Brilliant Violet 510 (clone GK1.5) | Figure 1C (DP, CD4/CD8 analysis), Figure 3B (DP, CD4/CD8 analysis), Figure 6A (Total Thymocytes sort and analysis), Figure 3-figure supplement 1 (Total Thymocytes analysis), Figure 3-figure supplement 2 (Total and Depleted Splenocytes analysis) |
| Anti-mouse CD8α APC (clone 53-6.7) | Figure 1C (DP, CD4/CD8 analysis), Figure 3B (DP, CD4/CD8 analysis), Figure 6A (Total Thymocytes sort and analysis), Figure 3-figure supplement 1 (Total Thymocytes analysis), Figure 3-figure supplement 2 (Total Splenocytes analysis), Figure 3-figure supplement 3 (Total Thymocytes and Splenocytes analysis), Figure 3-figure supplement 4 (Total Thymocytes analysis) |
| Anti-mouse TCRβ APC-eFluor 780 (clone H57-597) | Figure 6A (Total Thymocytes sort), Figure 3-figure supplement 1 (Total Thymocytes analysis) |
| Anti-mouse CD25 APC-eFluor 780 (clone PC61.5) | Figure 1C (DP, CD4/CD8 analysis), Figure 2 (BM-derived DNs sort), Figure 3B (DP, CD4/CD8 analysis), Figure 3D (Thymocyte DNs sort), Figure 5A (BM-derived DNs analysis), Figure 6A (Thymocyte DNs sort), Figure 6B (FL DNs analysis), Figure 3-figure supplement 1 (Total Thymocytes analysis), Figure 3-figure supplement 2,3 (Total Splenocytes analysis) |
| Anti-mouse CD19 eFluor 450 (clone 1D3/6D5) | Figure 3-figure supplement 2 (Total Splenocytes analysis) |
| Anti-mouse CD117 (cKit) APC (clone 2B8) | Figure 3D (Thymocyte DNs sort), Figure 6A (Thymocyte DNs sort) |
| Anti-mouse CD45 APC-eFluor 780 (clone 30-F11) | Figure 3D (Thymocyte DNs analysis), Figure 6A (Thymocyte DNs, DP, CD4SP analysis) |
| Anti-mouse CD25 APC (clone PC61.5) | Figure 6A (Thymocyte DNs analysis) |
| Anti-mouse CD4 APC-eFluor 780 (clone GK1.5) | Figure 6B (FL DNs sort), Figure 3-figure supplement 3 (Total Thymocytes and Splenocytes analysis) |
| Anti-mouse CD8α APC-eFluor 780 (clone 53-6.7) | Figure 6B (FL DNs sort), Figure 3-figure supplement 2 (Depleted Splenocytes analysis), Figure 3-figure supplement 3 (Total Splenocytes analysis) |
| Anti-mouse CD45 PerCP-Cyanine5.5 (clone 30-F11) | Figure 6B (FL DNs sort) |
| Anti-mouse CD45 APC (clone 30-F11) | Figure 6B (FL DNs analysis) |
| Anti-mouse CD5 eFluor 450 (clone 53-7.3) | Figure 3-figure supplement 1 (Total Thymocytes analysis) |
| Anti-mouse TCRγδ APC (clone GL3) | Figure 3-figure supplement 1 (Total Thymocytes analysis), Figure 3-figure supplement 2 (Depleted Splenocytes analysis), Figure 3-figure supplement 3 (Total Thymocytes and Splenocytes analysis) |
| Anti-mouse CD49b eFluor 450 (clone DX5) | Figure 3-figure supplement 1 (Total Thymocytes analysis) |
| Anti-mouse NK1.1 APC (clone PK136) | Figure 3-figure supplement 1 (Total Thymocytes analysis), Figure 3-figure supplement 2 (Depleted Splenocytes analysis) |
| Anti-mouse CD3ε APC-eFluor 780 (clone 145-2C11) | Figure 3-figure supplement 1 (Total Thymocytess analysis), Figure 3-figure supplement 2 (Depleted Splenocytes analysis), Figure 3-figure supplement 3 (Total Thymocytes and Splenocytes analysis) |
| Anti-mouse TCRβ eFluor 450 (clone H57-597) | Figure 3-figure supplement 2 (Depleted Splenocytes analysis), Figure 3-figure supplement 3 (Total Thymocytes and Splenocytes analysis) |
| Anti-mouse CD49b Biotin (clone DX5) | Figure 3-figure supplement 2 (Depleted Splenocytes analysis) |
| Anti-mouse CD62L APC (clone MEL-14) | Figure 3-figure supplement 2 (Depleted Splenocytes analysis), Figure 3-figure supplement 3 (Total Splenocytes analysis) |
| Anti-mouse CD45.2 Brilliant Violet (clone 104) | Figure 3-figure supplement 3 (Total Thymocytes and Splenocytes analysis) |
| Anti-mouse CD4 eFluor 450 (clone GK1.5) | Figure 3-figure supplement 3 (Total Splenocytes analysis), Figure 3-figure supplement 4 (Total Thymocytes analysis) |
| Anti-mouse CD45 eFluor 450 (clone 30-F11) | Figure 5A (BM-derived DNs analysis) |
| Streptavidin PerCP-Cyanine5.5 | Figure 2 (BM-derived DNs sort), Figure 3D (Thymocyte DNs sort) |
| Streptavidin Brilliant Violet 510 | Figure 6A (Thymocyte DNs sort and analysis), Figure 6B (FL DNs analysis), Figure 3-figure supplement 1 (Total Thymocytes analysis), Figure 3-figure supplement 2 (Depleted Splenocytes analysis) |
